# Labor induction in China: a nationwide survey

**DOI:** 10.1186/s12884-022-04760-6

**Published:** 2022-06-01

**Authors:** Jing Zhu, Lili Xue, Huaxiang Shen, Lin Zhang, Danni Lu, Yanlin Wang, Yu Zhang, Jun Zhang

**Affiliations:** 1 The International Peace Maternity and Child Health Hospital, Shanghai Jiao Tong University School of Medicine, Shanghai, China; 2 Ministry of Education-Shanghai Key Laboratory of Children’s Environmental Health, Xinhua Hospital, Shanghai Jiao Tong University School of Medicine, 1665 Kongjiang Rd, Shanghai, 200092 China; 3 Department of Obstetrics, Jiaxing University Affiliated Women and Children Hospital, Jiaxing, China; 4 Department of Obstetrics and Gynecology, Renji Hospital, Shanghai Jiao Tong University School of Medicine, 160 Pujian Rd, Shanghai, 200127 China

**Keywords:** Labor induction, Obstetrics, Epidemiology

## Abstract

**Background:**

Overmedicalization in labor management and delivery, including labor induction, is an increasing global concern. But detailed epidemiological data on labor induction in China remains unclear.

**Methods:**

This was a cross-sectional study of data (2015–2016) from 96 hospitals in 24 (of 34) Chinese administrative divisions. Multivariable logistic regression analysis was used to assess the association between medical conditions and cesarean delivery among women undergoing induction. Linear regression analysis was performed to assess the association between the prelabor cesarean delivery and labor-induction rates in each hospital. The impacts of labor induction and prelabor cesarean delivery on maternal and neonatal outcomes were compared in low-risk women.

**Results:**

Among 73 901 eligible participants, 48.1% were nulliparous. The overall weighted rate of labor induction in China was 14.2% (95% CI, 11.1–17.2%), with 18.4% (95% CI, 14.5–22.3%) in nulliparas and 10.2% (95% CI, 7.7–12.8%) in multiparas. Regardless of the induction method, the overall vaginal delivery rate was 72.9% (95% CI, 68.6–77.3%) in nulliparas and 86.6% (95% CI, 79.7–93.5%) in multiparas. Hospitals with a higher rate of nonmedically indicated cesarean delivery had a lower labor-induction rate in nulliparas (β =  − 0.57%; 95% CI, − 0.92 to − 0.22%; *P* = 0.002). Compared with prelabor cesarean delivery, labor induction in low-risk women was not associated with adverse maternal and neonatal outcomes.

**Conclusion:**

The 2015–2016 labor-induction rate in China was 18.4% in nulliparas and 10.2% in multiparas. The proportion of prelabor cesarean delivery may contribute to regional differences in the labor-induction rate. Compared with prelabor cesarean delivery, labor induction in low-risk women may not increase severe maternal and neonatal morbidity.

**Supplementary Information:**

The online version contains supplementary material available at 10.1186/s12884-022-04760-6.

## Introduction

Labor induction is a common clinical procedure. When the benefits of expeditious delivery outweigh the risks of continuing the pregnancy, labor induction is considered as a therapeutic and preventive option. To achieve vaginal delivery, various approaches have been used to stimulate uterine contractions before the spontaneous onset of labor. Such interventions may impact the health of women and their babies. Thus, the benefits and potential risks need to be clearly justified before inducing labor. The World Health Organization (WHO), the American College of Obstetricians and Gynecologists (ACOG), and the National Institute for Health and Care Excellence (NICE) in England all recommend that induction be performed with consideration of medical indications, maternal and fetal conditions, gestational age, and cervical status [1–3].

Unfortunately, as the prevalence of facility-based births increases, excessive, unnecessary, and even inappropriate use of obstetric interventions have become a concern in some high-income countries and a growing number of low- and middle-income countries [4]. Overmedicalization during childbirth may improve outcomes in certain circumstances, but may also be harmful and costly when used inappropriately. Examples include nonmedically indicated cesarean delivery, routine episiotomy, high rates of labor induction, and augmentation [5–7]. Hence, epidemiological studies are warranted to reveal national or regional coverage rates of obstetric interventions and examine medical indications for the procedures, so that strategies could be taken to ensure that all women receive evidence-based maternity care.

There is a growing divergence in labor induction rates worldwide. In 2004–2005, one in every five deliveries in the UK was induced, while induction was used in 42.9% of nulliparous women and 31.8% of multiparous women in the US in 2002–2008 [3, 8]. In Brazil, the labor-induction rate increased to 43.0% in 2004, with a simultaneous increase in the cesarean delivery rate to 43.2% [9]. In contrast, labor induction is still less common in Africa and Asia, where induction accounted for 4.4% of total births in 2004–2005 and 12.1% in 2007–2008, respectively [10]. Anecdotal evidence suggests that the prevalence of labor induction in China was low before 2011, estimated as 6.4% in 2007–2008 and 7.0% in 2010–2011[4]. Given the high rate of prelabor cesarean delivery and the relatively low rate of labor induction in China [11], we wondered whether labor induction in low-risk women should be considered as an alternative to cesarean delivery upon maternal request. However, in-depth analyses of more recent data on indications for, and methods and the success rate of induction in China are lacking. Thus, we used data from the China Labor and Delivery Survey with the aim to separately describe the patterns of labor induction in nulliparous and multiparous women, and to dissect the impacts of labor induction and prelabor cesarean delivery on maternal and neonatal outcomes.

## Methods

### Study design

The China Labor and Delivery Survey was a nationwide cross-sectional study conducted from March 1, 2015 to December 31, 2016. The participating hospitals were solicited through obstetric networks. Hospitals with 1000 or more deliveries per year were eligible for inclusion. Depending on the annual delivery volume of the hospitals, 5–10 consecutive weeks were randomly selected in a 12-month period as the study window. Within the selected weeks, all births at ≥ 24 weeks of gestation or with a birthweight of ≥ 500 g were included. We obtained anonymized data from participants’ medical records; information on maternal sociodemographic characteristics, medical and pregnancy histories, pregnancy and labor complications, and perinatal outcomes was extracted by trained staff. Criteria for data extraction were defined in an operations manual that was used for staff training and monitoring of data collection. The completed data-extraction forms were reviewed by the data manager for completeness before they were entered into the database. Methodological details on sampling, data extraction, and data management have been published elsewhere [12, 13].

A total of 96 hospitals distributed in 24 (out of 34) provinces, autonomous regions and municipalities in China were included in the analysis. This study was approved by the Ethics Review Board of the Xinhua Hospital Affiliated to the Shanghai Jiao Tong University School of Medicine (XHEC–C–2015–006), the Research Project Review Panel (RP2) of the UNDP/UNFPA/UNICEF/WHO/World Bank Special Programme of Research, Development and Research Training in Human Reproduction, at the Department of Sexual and Reproductive Health and Research at the World Health Organization, by the WHO Research Ethics Review Committee (HRP Study A65899) and participating hospitals.

### Definitions

Labor induction was defined as the process of artificial stimulation of the uterus to start labor [14]. A woman was considered to have undergone labor induction if an induction, or the method or start time thereof was recorded before the onset of labor. Gestational age was ascertained on the basis of the last menstrual period, or by ultrasound dating in the first trimester if the date of the last menstrual period was uncertain. Standard partitioning of geographical regions in China (East, North, South, Central, Northeast, Northwest, and Southwest) was used to reveal regional differences [15]. Hospital levels were determined by the Chinese Ministry of Health based on the number of beds, categories of clinical departments, numbers of medical staff, type and quantity of equipment, and hospital funding [16]. Labor analgesia included epidural analgesia and other relaxation techniques for pain management.

Indications for labor induction included gestational hypertension, preeclampsia/eclampsia, gestational diabetes, premature rupture of membranes (PROM), late-term and post-term pregnancies, fetal death, maternal medical complications (e.g., diabetes mellitus, renal disease, autoimmune disease), and fetal conditions (e.g., small for gestational age [SGA], abnormal antenatal testing results, fetal anomalies). Late-term pregnancy was defined as a pregnancy reaching 41–41^+6^ weeks of gestation, whereas a post-term pregnancy was defined as a pregnancy reaching or exceeding 42 weeks of gestation [17]. SGA was determined as a birthweight less than the 10th percentile for a given gestational week based on a global reference for fetal-weight and birthweight percentiles [18]. Macrosomia was defined as a birthweight of ≥ 4000 g, regardless of the gestational age [19]. Abnormal or indeterminate fetal heart rate tracings or abnormal biophysical profiles were considered abnormal antenatal testing results. An induction performed when there were no maternal or fetal medical conditions or obstetric complications, while the gestational age was less than 41 weeks, was categorized as nonmedically indicated. A uterine scar could be due to either a previous cesarean delivery or other uterine surgery.

We used a simplified Bishop score, comprised of cervical dilation, effacement, and fetal station, to assess cervical readiness for induction. A simplified Bishop score ≤ 4 was considered indicative of an unripe cervix, which has a similar sensitivity and specificity to an original Bishop score ≤ 6, the definition of an unfavorable cervix [20]. Methods of induction were grouped into artificial rupture of membranes, mechanical methods, and use of prostaglandin and oxytocin. We did not exclude any births based on the method used for cervical ripening and labor induction. The attempted mode of delivery was recorded in the medical records when women were admitted to hospitals, as one of the following: spontaneous labor, labor induction, cesarean delivery without indications, cesarean delivery with indications, and unknown.

We further compared labor induction and prelabor cesarean delivery in low-risk women on maternal and neonatal outcomes. Low-risk was defined as term pregnancies without any of the following maternal or fetal medical conditions or obstetrical complications: chronic hypertension, diabetes mellitus, thyroid disease, renal disease, autoimmune disease, heart disease, gestational hypertension, preeclampsia/eclampsia, gestational diabetes, cholestasis, SGA, suspected macrosomia, abnormal antenatal testing results, antenatal stillbirth, fetal anomaly, breech or other non-cephalic presentation, PROM, late-term or post-term pregnancy, uterine scar, placental abruption, placenta previa, and prolapse of the cord.

### Statistical analysis

Each birth was assigned a weight with inverse probability weighting, taking into account the number of births in the same administrative region in hospitals of the same level, the total number of births in the prior year in the same hospital, and the number of records reviewed in the same hospital. The 2016 China Statistical Yearbook was used to determine the number of deliveries in each administrative region [15].

We used frequencies to describe induction rates for each maternal characteristic, medical condition, initial cervical assessment, and method of induction among women undergoing labor induction. Frequencies were calculated separately for nulliparous and multiparous women by using the PROC SURVEYFREQ procedure in SAS. Multivariable logistic regression was used to assess the association between medical conditions and cesarean delivery among women undergoing labor induction, by using the PROC SURVEYLOGISTIC procedure in SAS. Both procedures incorporated the sampling weight and the clustering of births within hospitals. We reported crude and adjusted odds ratios (ORs) with 95% confidence intervals (CIs) in nulliparous and multiparous women, controlling for maternal age, race, insurance status, education level, body mass index (BMI) at delivery, number of fetuses, fetal presentation, use of labor analgesia, hospital location, and hospital level. A weighted proportion of attempted mode of delivery, i.e., spontaneous labor, labor induction, cesarean delivery without indications, and cesarean delivery with indications, was calculated to reveal the differences between geographical regions. Linear regression analysis was performed to assess the association between the prelabor cesarean delivery and labor-induction rates in each hospital, adjusting for sampling weight, hospital level, and hospital location. The impacts of labor induction and prelabor cesarean delivery on maternal and neonatal outcomes were compared in low-risk women. SAS version 9.4 (SAS Institute Inc., Cary, NC) was used for all statistical analyses.

## Results

**Fig. 1:**
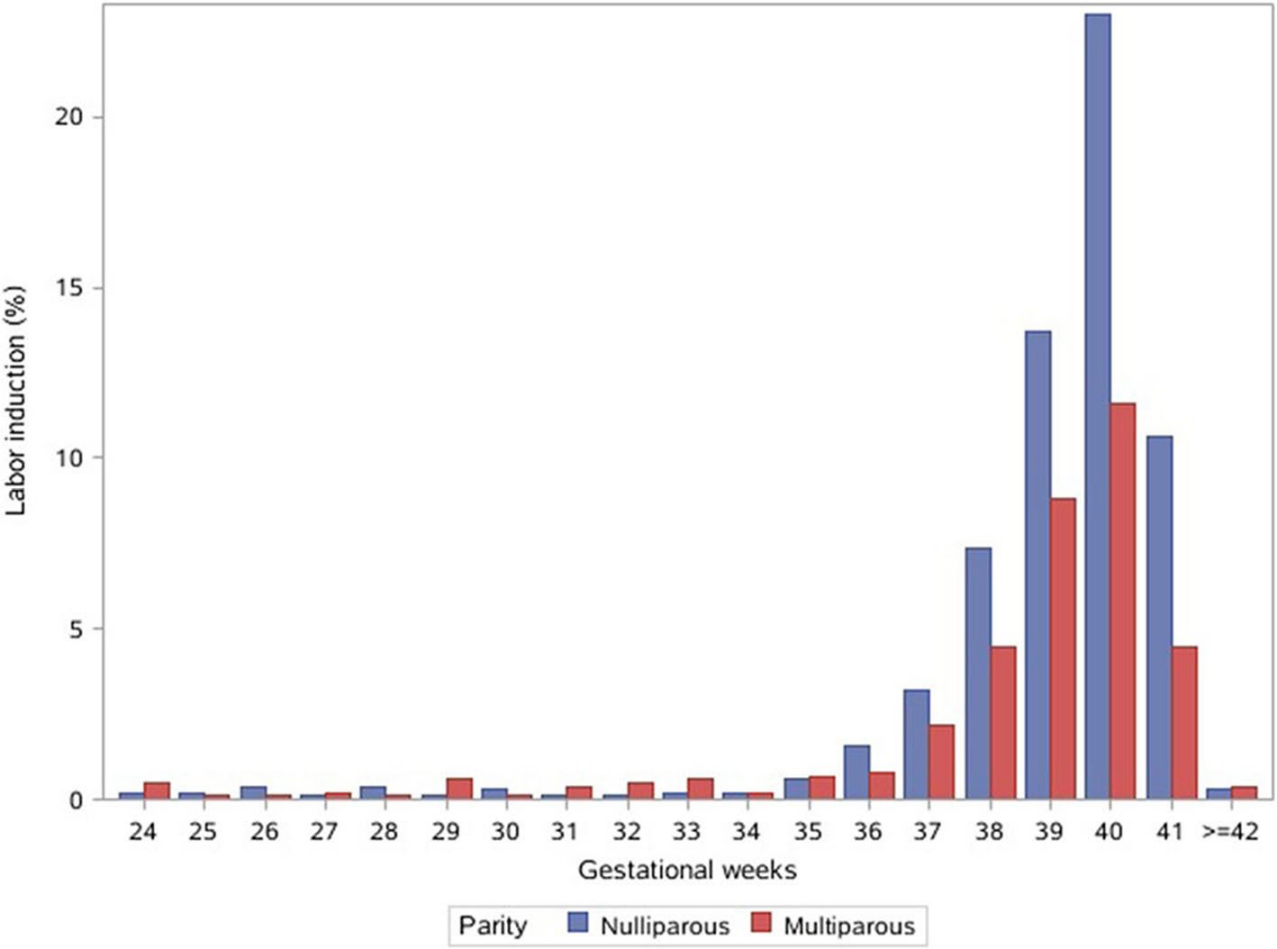
Distribution of labor induction by weeks of gestation

We collected data on 75 132 deliveries during the study period, yielding a response rate of 96.6% (75 132/77 789). We excluded 319 participants with unknown parity and 912 women with unknown method of delivery, leaving 73 901 deliveries for the analysis. Of these women, 48.1% were nulliparous. The overall weighted rate of labor induction in China was 14.2% (95% CI, 11.1–17.2%) in 2015 and 2016, with 18.4% (95% CI, 14.5–22.3%) in nulliparas and 10.2% (95% CI, 7.7–12.8%) in multiparas. The distribution of women undergoing labor induction by weeks of gestation is illustrated in Fig. 1. The induction rate peaked at 40 weeks and decreased thereafter.

Table 1 presents the prevalence of labor induction based on maternal and clinical characteristics. The association between sociodemographic characteristics and cesarean delivery in women with induced labor is presented in Additional file Table S1. In nulliparas, labor induction increased with higher maternal education level and BMI at delivery, and was more prevalent among women with social health insurance compared with those without. The prevalence of labor induction was 18.8% in singleton pregnancies and 3.2% in multiple gestations, among nulliparas. In multiparas, the induction rate varied little according to maternal education level, social health insurance status, and BMI at delivery. However, maternal overweight and obesity were associated with an increased risk of intrapartum cesarean delivery in both nulliparous and multiparous women who underwent labor induction.

**Table 1:** Prevalence of women undergoing labor induction in China

Characteristics	Nulliparas		Multiparas	
	Proportion of women, %^a^, *n* = 4 290 936	Prevalence of labor induction, % (95% CI)^a^	Proportion of women, %^a^, *n* = 4 635 107	Prevalence of labor induction, % (95% CI)^a^
Maternal age (years)				
< 25	25.7	13.6 (10.0–17.2)	15.8	12.5 (7.5–17.5)
25–29	54.9	19.7 (16.1–23.4)	37.1	9.5 (6.6–12.4)
30–34	15.9	22.1 (14.2–29.9)	29.4	11.2 (7.1–15.2)
≥ 35	3.5	16.7 (8.3–25.1)	17.7	8.3 (6.1–10.5)
Race				
Han	97.3	18.4 (14.5–22.4)	96.2	10.3 (7.7–12.9)
Other	2.7	17.6 (11.0–24.1)	3.8	8.8 (4.3–13.4)
Education (years)				
Less than high school (≤ 9)	23.0	13.4 (8.9–17.9)	51.5	10.4 (7.3–13.5)
High school (10–12)	20.1	19.5 (15.3–23.7)	20.7	12.0 (8.0–16.0)
College and above (> 12)	56.9	21.2 (16.3–26.0)	27.8	9.9 (7.0–12.8)
Social health insurance				
Yes	67.5	20.1 (15.1–25.1)	55.9	11.5 (7.9–15.1)
No	32.5	15.3 (12.1–18.5)	44.1	9.0 (6.8–11.1)
BMI at delivery (kg/m^2^)				
< 18.5	0.5	4.7 (0.3–9.1)	0.4	4.6 (0.0–9.4)
18.5–23.9	17.0	16.6 (12.4–20.9)	14.1	8.3 (5.8–10.9)
24.0–27.9	41.7	17.1 (13.9–20.3)	37.5	10.3 (7.2–13.4)
≥ 28.0	29.8	20.7 (15.8–25.6)	31.0	8.7 (6.2–11.2)
Unknown	11.0	20.7 (13.5–27.8)	17.0	14.4 (9.2–19.7)
Number of fetuses				
Singleton	97.8	18.8 (14.7–22.8)	98.0	10.4 (7.7–13.0)
Multiple	2.2	3.2 (0.0–6.4)	2.0	3.5 (0.4–6.6)
Fetal presentation				
Cephalic	96.0	19.1 (14.9–23.3)	97.1	10.4 (7.8–13.0)
Breech or other non-cephalic	4.0	1.7 (0.4–3.0)	2.9	5.2 (0.7–9.7)
Gestational age at delivery (weeks)				
< 37	7.5	18.1 (9.5–26.6)	7.6	18.7 (11.3–26.0)
≥ 37	92.5	18.6 (14.9–22.3)	92.4	9.6 (7.4–11.8)
Labor analgesia				
Yes	28.8	20.3 (12.6–27.9)	26.6	11.6 (7.4–15.8)
No	71.2	17.7 (14.6–20.8)	73.4	9.7 (7.6–11.8)
Hospital level				
Level 2	46.0	15.8 (11.4–20.2)	61.0	9.8 (6.6–12.9)
Level 3	54.0	20.6 (15.2–26.0)	39.0	11.0 (6.8–15.1)

*BMI* Body mass index, *CI* Confidence interval

^a^The PROC SURVEYFREQ procedure was used in SAS to calculate these frequencies, adjusting for sampling weight and clustering of births within hospitals

Table 2 presents the simplified Bishop score at the start of induction and the methods of induction. Approximately 26.2% of nulliparas and 23.4% of multiparas had favorable cervixes before induction. Oxytocin was the most common method of induction, used in 79.9% of nulliparas and 76.5% of multiparas, followed by artificial rupture of membranes. Prostaglandins and mechanical methods were used less commonly in both groups. Regardless of the induction method, the overall vaginal delivery rate was 72.9% (95% CI, 68.6–77.3%) in nulliparas and 86.6% (95% CI, 79.7–93.5%) in multiparas.

**Table 2:** Initial simplified Bishop score and method of induction among women undergoing labor induction in China

	Nulliparas	Multiparas
	Proportion of women, %^a^, *n* = 790 253	Proportion of women, %^a^, *n* = 473 866
Initial cervical dilation (cm)		
Closed	66.6	70.9
1–2	32.2	28.0
3–4	1.2	1.1
5–6	0	0
Initial cervical effacement (%)		
0–30	25.1	37.6
40–50	12.6	13.5
60–70	19.2	13.2
80–100	43.1	35.7
Initial station		
− 3	42.1	54.8
− 2	44.4	32.9
− 1 or 0	10.4	9.0
+ 1 or + 2	3.1	3.3
Simplified Bishop score ≥ 5	26.2	23.4
Method of induction		
Artificial rupture of membranes	25.3	18.9
Prostaglandins	9.5	9.9
Mechanical	8.9	6.9
Oxytocin	79.9	76.5

^a^The PROC SURVEYFREQ procedure was used in SAS to calculate these frequencies, adjusting for sampling weight and clustering of births within hospitals

**Fig. 2:**
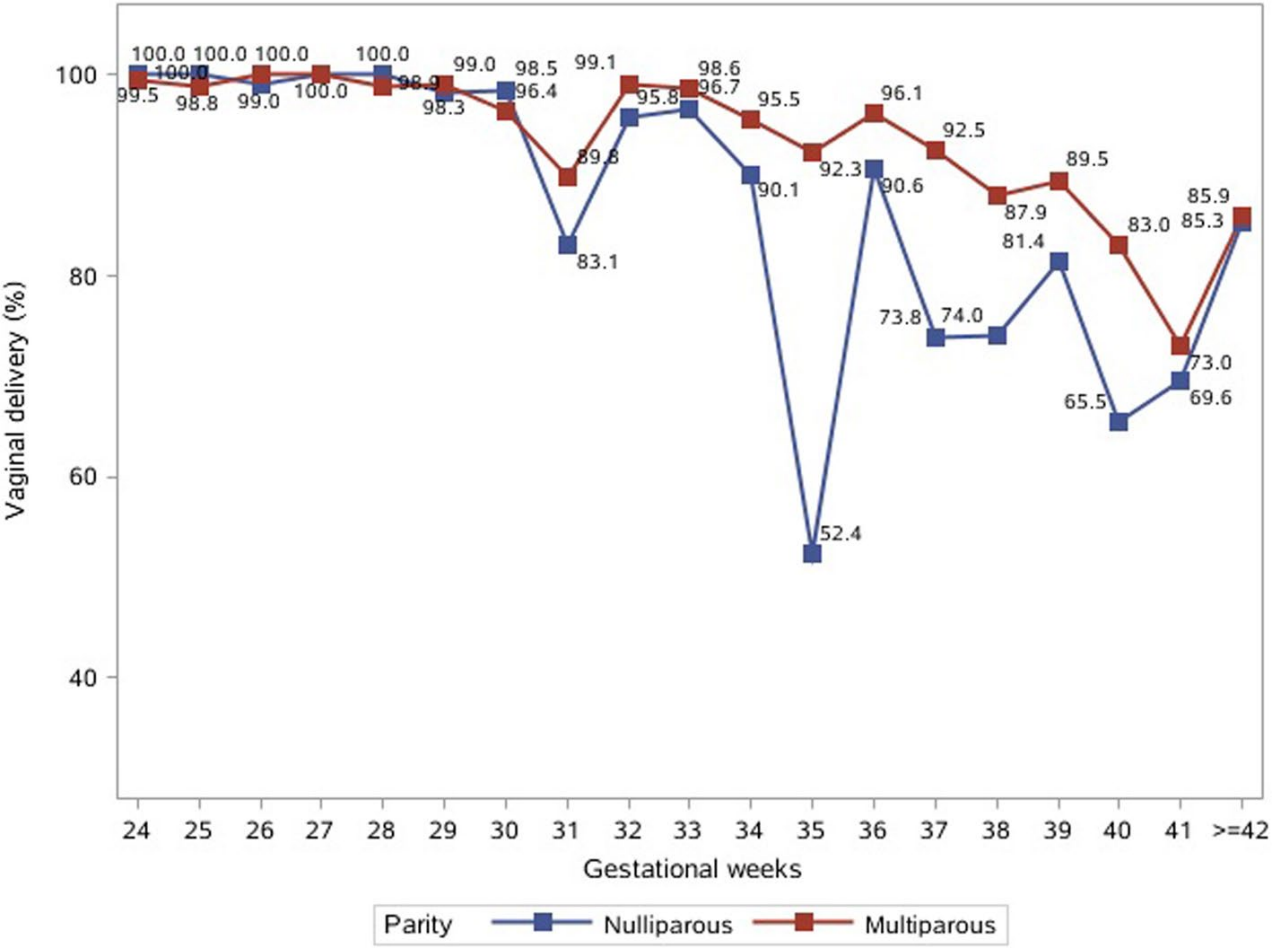
Vaginal delivery rate among women undergoing labor induction

Figure 2 illustrates the vaginal delivery rates among nulliparous and multiparous women undergoing labor induction by weeks of gestation. Both nulliparas and multiparas achieved a high vaginal-delivery rate (> 95%) before 30 weeks of gestation, and the rate generally declined and diverged with advancing weeks of gestation. At 40 weeks of gestation, the rates of vaginal delivery were 65.5 and 83.0% in nulliparas and multiparas, respectively.

**Fig. 3:**
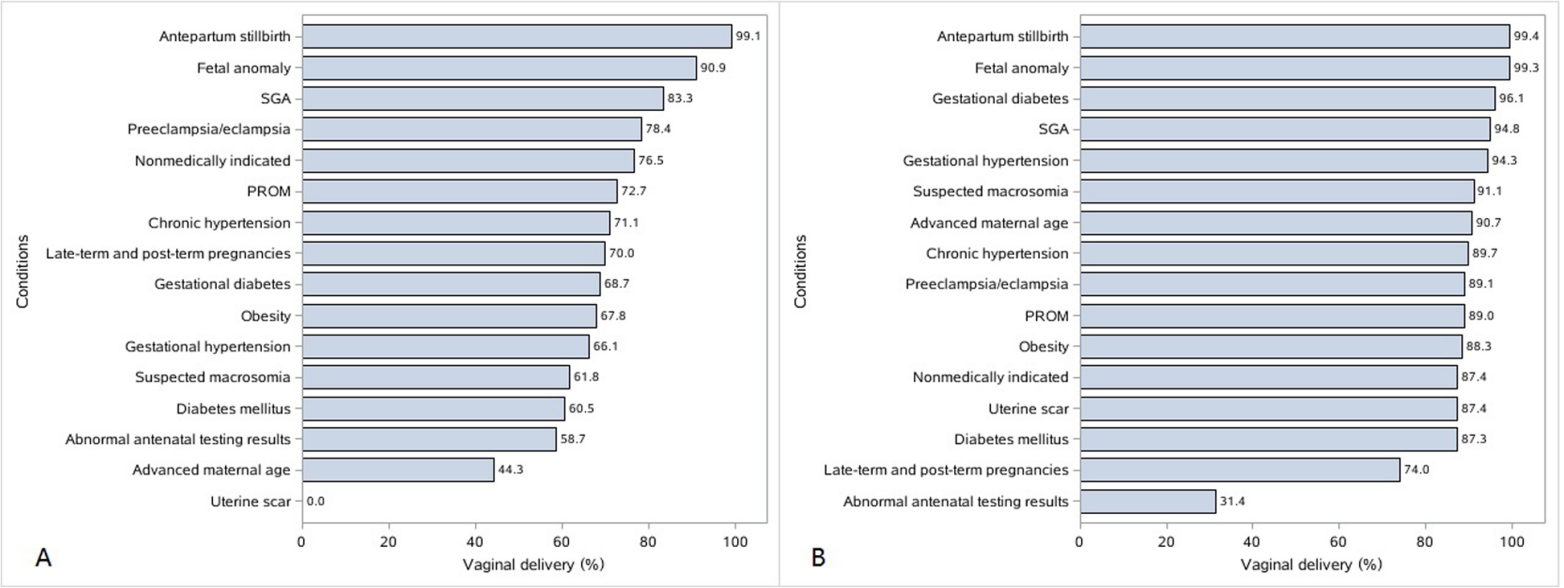
Vaginal delivery rate among women undergoing labor induction with various medical indications. **A** Nulliparous women. **B** Multiparous women. SGA, small for gestational age; PROM, premature rupture of membrane

Table 3 summarizes the prevalence of labor induction by indications and the association between medical indications and cesarean delivery in women undergoing labor induction, compared with women undergoing induction without such an indication. The vaginal-delivery rate after labor induction according to indications and the median gestational age at delivery are provided in Fig. 3 and Additional file Table S2. Women with gestational diabetes had the highest induction rate: 30% in nulliparas and 17.3% in multiparas. Nulliparous women with gestational hypertension, preeclampsia/eclampsia, or chronic hypertension had induction rates of 13.4%, 17.0%, and 19.0%, respectively. Abnormal antenatal testing results were associated with an increased risk of cesarean delivery in both nulliparous and multiparous women. The prevalence of labor induction for PROM was 29.9 and 27.8% in nulliparas and multiparas, respectively. For late-term and post-term pregnancies, 40.7% of nulliparous women and 22.1% of multiparous women underwent labor induction. Compared with women undergoing labor induction before 41 weeks of gestation, a significantly increased cesarean delivery rate was observed in late-term and post-term pregnancies, with an adjusted OR of 1.48 (95% CI, 1.10–1.99) for nulliparas and 2.87 (95% CI, 1.70–4.84) for multiparas. In multiparas, 91.3% of uterine scars were due to previous cesarean sections. We noticed that relatively few women with uterine scars underwent labor induction, and the corresponding rate of vaginal delivery was 0% in nulliparas and 87.4% in multiparas. Approximately 12.7% of nulliparas and 7.9% of multiparas underwent labor induction without medical indications.

**Table 3:** Indications and risks for cesarean section among women undergoing labor induction in China

	Nulliparas				Multiparas			
	Proportion of women	Prevalence of labor induction	Cesarean delivery after labor induction		Proportion of women	Prevalence of labor induction	Cesarean delivery after labor induction	
	%^a^, *n* = 4 290 936	% (95% CI)^a^	Crude OR (95% CI)^a^	Adjusted OR (95% CI)^a b^	%^a^,*n* = 4 635 107	% (95% CI)^a^	Crude OR (95% CI)^a^	Adjusted OR (95% CI)^a b^
Maternal complications								
Chronic hypertension	0.2	19.0 (7.1–30.9)	1.10 (0.46–2.62)	0.88 (0.36–2.13)	0.6	16.0 (0.0–34.2)	0.74 (0.12–4.58)	1.79 (0.28–11.3)
Gestational hypertension	1.6	13.4 (7.6–19.1)	1.39 (0.63–3.07)	1.04 (0.46–2.34)	1.6	5.2 (1.9–8.6)	0.39 (0.13–1.19)	0.34 (0.08–1.48)
Preeclampsia/eclampsia	2.4	17.0 (8.6–25.4)	0.74 (0.24–2.24)	0.79 (0.28–2.28)	2.3	8.5 (2.4–14.7)	0.79 (0.21–2.92)	1.27 (0.43–3.73)
Diabetes mellitus	0.9	11.9 (5.2–18.6)	1.77 (0.78–4.01)	1.89 (0.77–4.60)	1.0	9.1 (3.7–14.4)	0.94 (0.15–5.93)	1.45 (0.17–12.4)
Gestational diabetes	11.5	30.0 (18.8–41.2)	1.29 (0.85–1.96)	1.11 (0.59–2.07)	9.5	17.3 (7.1–27.4)	0.23 (0.06–0.85)	0.31 (0.11–0.93)
Other conditions^c^	2.0	15.8 (8.1–23.5)	0.63 (0.25–1.59)	0.40 (0.12–1.41)	1.5	9.0 (1.1–16.9)	0.36 (0.10–1.26)	0.77 (0.23–2.54)
Fetal indications								
SGA (< 10^th^ percentile)	11.2	15.5 (11.5–19.6)	0.50 (0.27–0.94)	0.55 (0.30–1.01)	9.0	16.1 (8.7–23.5)	0.32 (0.08–1.23)	0.30 (0.06–1.64)
Suspected macrosomia	8.5	22.4 (15.3–29.5)	1.78 (1.28–2.47)	1.56 (0.92–2.66)	9.0	9.1 (5.7–12.5)	0.61 (0.28–1.33)	0.41 (0.18–0.95)
Abnormal antenatal testing results	5.2	19.7 (15.1–24.3)	1.98 (1.16–3.37)	1.97 (1.06–3.68)	3.0	15.3 (9.8–20.8)	18.2 (6.9–48.0)	28.2 (8.8–90.0)
Antepartum stillbirth	0.7	60.2 (52.7–67.8)	0.03 (0.01–0.09)	0.02 (0.01–0.11)	1.3	63.1 (39.8–86.5)	0.04 (0.01–0.15)	0.02 (0.00–0.11)
Fetal anomalies	0.5	37.9 (24.0–51.8)	0.27 (0.07–1.02)	0.35 (0.10–1.30)	1.0	38.4 (28.6–48.3)	0.04 (0.01–0.25)	0.07 (0.01–0.52)
PROM	14.3	29.9 (23.1–36.6)	1.01 (0.83–1.24)	1.03 (0.81–1.31)	11.1	27.8 (19.2–36.4)	0.73 (0.36–1.49)	0.63 (0.36–1.10)
Late-term and post-term pregnancies	7.9	40.7 (31.5–49.8)	1.19 (0.95–1.49)	1.48 (1.10–1.99)	6.0	22.1 (15.9–28.3)	2.70 (1.55–4.70)	2.87 (1.70–4.84)
Uterine scar	0.8	0.5 (0.0–1.2)	NA	NA	34.0	1.9 (1.1–2.8)	0.93 (0.27–3.23)	1.37 (0.30–6.25)
Nonmedically indicated^d^	47.1	12.7 (10.0–15.5)	0.76 (0.53–1.10)	0.76 (0.46–1.27)	34.4	7.9 (5.7–10.0)	0.91 (0.25–3.35)	0.89 (0.22–3.56)

*SGA* Small for gestational age, *PROM* Premature rupture of membrane, *OR* Odds ratio, *CI* Confidence interval, *NA* Not applicable

^a^The PROC SURVEYFREQ procedure was used in SAS to calculate these frequencies, adjusting for sampling weight and clustering of births within hospitals

^b^The PROC SURVEYLOGISTIC procedure was used in SAS to evaluate the association between medical indications and cesarean section in women undergoing labor induction, compared with women undergoing induction without such an indication. We adjusted for maternal age, race, insurance status, education level, body mass index at delivery, number of fetuses, fetal presentation, labor analgesia, hospital location, and hospital level

^c^Other conditions included: thyroid disease, renal disease, and autoimmune disease

^d^Nonmedically indicated: women without the following conditions: chronic hypertension, diabetes mellitus, thyroid disease, renal disease, autoimmune disease, heart disease, gestational hypertension, preeclampsia/eclampsia, gestational diabetes, cholestasis, SGA, suspected macrosomia, abnormal antenatal testing results, antenatal stillbirth, fetal anomaly, breech or other non-cephalic presentation, PROM, late-term or post-term pregnancy, uterine scar, placental abruption, placenta previa, and prolapse of the cord

**Fig. 4:**
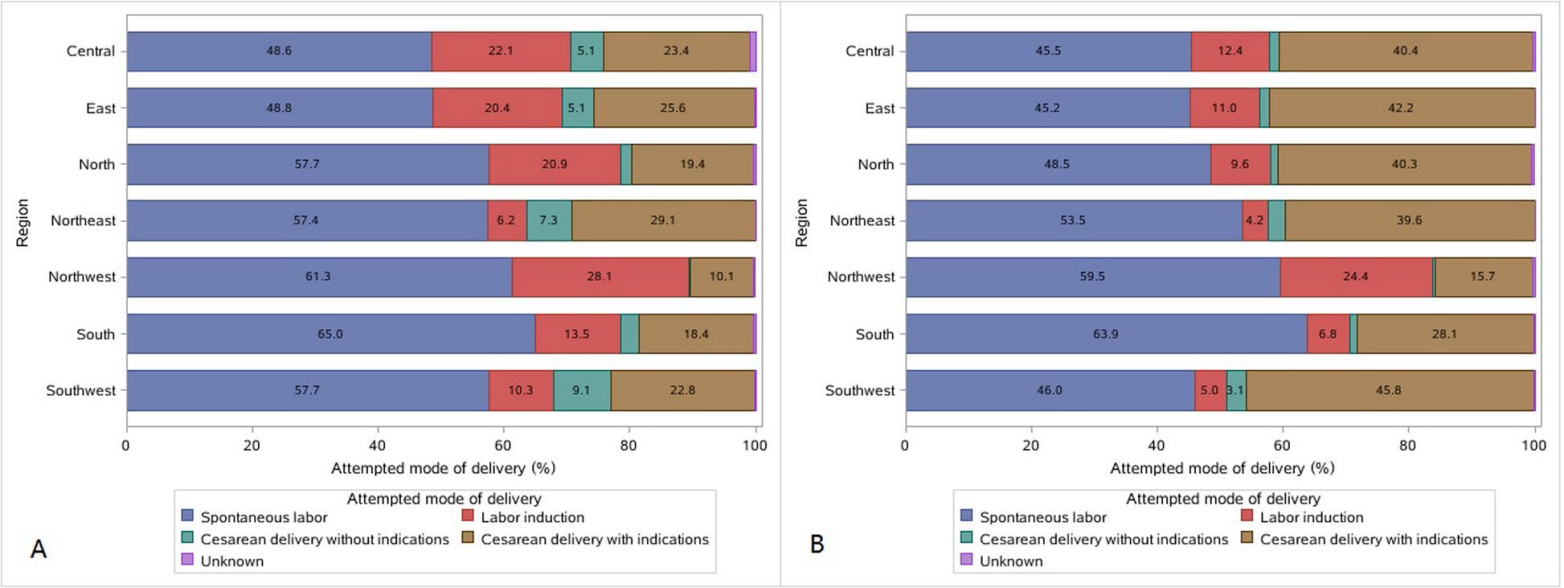
Attempted mode of delivery by geographical regions in China. **A** Nulliparous women. **B** Multiparous women

Figure 4 and Additional file Table S3 present the weighted proportion of attempted mode of delivery according to geographical region and parity. Both Northeast and Southwest had lower induction rates in nulliparas, as women in these two regions were more likely to undergo cesarean delivery without indications (7.3% in Northeast and 9.1% in Southwest). In contrast, nulliparous women in Northwest were less likely to undergo cesarean delivery (0.3% without and 10.1% with medical indications), resulting in higher rates of spontaneous (61.3%) and induced (28.1%) labor. Moreover, at the hospital level, a higher rate of cesarean delivery without medical indications was significantly associated with a lower rate of labor induction in nulliparas (β =  − 0.57%; 95% CI, − 0.92% to − 0.22%; *P* = 0.002) (Additional file Table S4).

We further compared the effects of labor induction and prelabor cesarean delivery in low-risk women on maternal and neonatal outcomes (Table 4). Low-risk pregnancies accounted for 40.5% of the study population. 75.2%, 10.6% and 14.0% of these women underwent spontaneous labor, induced labor and prelabor cesarean delivery, respectively. Characteristics of women undergoing labor induction and prelabor cesarean delivery are presented in Additional file Table S5. Overall, there was no substantial difference between these two groups of women. The rate of failed induction leading to cesarean delivery was 3.2 and 1.3% in nulliparas and multiparas, respectively. Despite that an increased rate of birth trauma was observed in induced labor, labor induction in low-risk women, compared with prelabor cesarean delivery, was not associated with adverse maternal and neonatal outcomes.

**Table 4:** Comparison of labor induction and prelabor cesarean delivery in low-risk women on maternal and neonatal outcomes

	Nulliparas				Multiparas			
	Labor induction (*n* = 257 145)	Prelabor cesarean delivery (*n* = 366 975)	Crude OR	Adjusted OR	Labor induction (*n* = 125 402)	Prelabor cesarean delivery (*n* = 139 101)	Crude OR	Adjusted OR
	N (%)^a^	N (%)^a^	(95% CI)^a^	(95% CI)^a b^	N (%)^a^	N (%)^a^	(95% CI)^a^	(95% CI)^a b^
Maternal outcomes								
Postpartum hemorrhage	8640 (3.4)	14,596 (4.0)	0.84 (0.43–1.64)	1.26 (0.76–2.08)	1922 (1.5)	1721 (1.2)	1.25 (0.45–3.51)	0.96 (0.36–2.58)
Postpartum infection	330 (0.1)	482 (0.1)	0.98 (0.29–3.34)	1.20 (0.34–4.27)	0 (0)	38 (0.0)	NA	NA
Amniotic fluid embolism	0 (0)	0 (0)	NA	NA	0 (0)	0 (0)	NA	NA
Admission to intensive care unit	975 (0.4)	1367 (0.4)	1.02 (0.30–3.48)	0.62 (0.16–2.37)	62 (0.0)	562 (0.4)	0.12 (0.01–1.06)	0.07 (0.01–3.29)
Neonatal outcomes								
Birth trauma	1456 (0.6)	132 (0.0)	15.9 (4.1–61.5)	9.1 (2.2–37.0)	138 (0.1)	0 (0)	NA	NA
Apgar score ≤ 7 at 5 min	833 (0.3)	1733 (0.5)	0.69 (0.16–2.92)	1.84 (0.40–8.48)	56 (0.0)	956 (0.7)	0.07 (0.01–0.48)	0.07 (0.01–0.63)
Admission to neonatal intermediate or intensive care unit	16,280 (6.3)	13,606 (3.7)	1.76 (0.56–5.48)	1.85 (0.54–6.33)	6254 (5.0)	7560 (5.4)	0.91(0.25–3.32)	0.96 (0.32–2.90)

*OR* Odds ratio, *CI* Confidence interval, *NA*, Not applicable

^a^The PROC SURVEYFREQ procedure was used in SAS to calculate these frequencies, adjusting for sampling weight and clustering of births within hospitals

^b^The PROC SURVEYLOGISTIC procedure was used in SAS to evaluate the association between labor induction and prelabor cesarean delivery in low-risk women on maternal and neonatal outcomes. We adjusted for maternal age, race, insurance, education, BMI at delivery, number of fetuses, hospital location and hospital levels

Low-risk women were women with term pregnancies and without the following conditions: chronic hypertension, diabetes mellitus, thyroid disease, renal disease, autoimmune disease, heart disease, gestational hypertension, preeclampsia/eclampsia, gestational diabetes, cholestasis, SGA, suspected macrosomia, abnormal antenatal testing results, antenatal stillbirth, fetal anomaly, breech or other non-cephalic presentation, PROM, late-term or post-term pregnancy, uterine scar, placental abruption, placenta previa, and prolapse of the cord

## Discussion

Our study, including deliveries from 96 hospitals across China, revealed an overall labor-induction rate of 14.2% in 2015–2016, with 18.4% in nulliparas and 10.2% in multiparas. A quarter of the women had favorable cervixes before induction. Over three-quarters received oxytocin as the method of labor induction. A total of 72.9% of nulliparous and 86.6% of multiparous women who underwent labor induction achieved vaginal delivery. The regional difference in labor-induction rates was observed in China. A higher proportion of prelabor cesarean delivery was associated with a lower rate of labor induction. Compared with prelabor cesarean delivery, labor induction in low-risk women was not associated with severe maternal and neonatal morbidity.

Trends of increasing use of labor induction have been reported in high-income countries since the 1990s. In the US, the induction rate rose from 9.5 to 14.9% from 1989 to 1998, and reached 23.1% in 2011 [21, 22]. In Australia, the rate of induction increased from 25.3 to 29.1% during 1998–2007 [23]. The UK and Canada had a similarly increasing trend [24]. In middle-income countries, limited data revealed an uneven coverage rate of labor induction, ranging from 1.8% in Paraguay to 71.0% in Iran during 2010–2012 [4]. The rate of induction continued to increase in Brazil (from 2.5 to 38.6%) from 1982 to 2011 [4, 9]. However, there has been a lack of epidemiological data on labor induction in China. Vogel and colleagues [10] used the WHO Global Survey dataset to estimate the rate of labor induction in China as 6.4% in 2007–2008, with 67.9% of those women achieving vaginal delivery. The induction rate in China was estimated to be around 7.0% in 2010–2011 [4]. Yet, these data do not distinguish between nulliparous and multiparous women. Given that the indications for labor induction, labor progression, and outcomes differ substantially between these two groups of women, we conducted stratified analyses to explore the utilization of this procedure.

The purpose of labor induction is to achieve vaginal delivery without compromising maternal and neonatal health. To assess the effectiveness of labor induction, cesarean delivery is most often used as a primary outcome in observational studies and clinical trials. In comparison with expectant management awaiting spontaneous labor, labor induction in uncomplicated singleton pregnancies reportedly reduces the risk of cesarean delivery and perinatal mortality [25–28]. Even in nulliparous women with an unfavorable cervix, labor induction did not result in statistically significant differences of most clinical outcomes [29]. The synthesized vaginal-delivery rate for women who underwent labor induction in uncomplicated singleton pregnancies was 73.6% for nulliparas and 81.3% for all women [26, 27]. Our findings were comparable with these results, as labor induction yielded a vaginal-delivery rate of 76.5% and 87.4% in uncomplicated pregnancies of nulliparas and multiparas, respectively, with a median gestational age at delivery of 39.9 weeks.

Our data, along with those of other studies [11, 30], suggest a distinct divergence in the initiation of labor between China and certain other countries. China tends to have a high prevalence of prelabor cesarean delivery, while countries such as the US and the UK use labor induction instead in women without medical indications. Unlike planned cesarean deliveries, vaginal deliveries after labor induction may occur at any time, and greater healthcare resources are required, including trained staff, medical procedures, and prolonged hospital stays. As most births are assisted by obstetricians in China, prelabor cesarean delivery is often considered as a safe, convenient and resource-saving approach in hospitals with a high volume of deliveries, when compared with labor induction. Further, as the cesarean delivery rate in China has been high during the last two decades, many obstetricians are more skilled in performing cesarean delivery than assisted vaginal deliveries. Fear of lawsuits for complications and malpractice in vaginal delivery also causes reluctance in Chinese obstetricians in terms of labor induction. Since China has implemented a universal two-child policy, more women are expected to have a subsequent pregnancy [31]. Primary cesarean deliveries should be decreased to reduce the overall cesarean delivery rate down the road. Hence, physician-oriented interventions, such as implementation of clinical practice guidelines combined with a mandatory second opinion for indications for cesarean delivery, and regular training in the use of forceps- and/or vacuum-assisted delivery, may reduce the cesarean delivery rate [32]. As our data showed that labor induction as an alternative to prelabor cesarean delivery might be considered, especially for low-risk nulliparous women.

Except for clinician’s beliefs and attitudes, maternal request for cesarean delivery may also influence the clinical practice. China is one of the countries that has a high rate of nonmedically indicated cesarean delivery, even after the onset of labor. In 2014, the Chinese Obstetricians and Gynecologists Association issued an expert consensus on cesarean delivery operations, which gave doctors the right to refuse a cesarean section upon maternal request [33]. However, some women still seek cesarean delivery as a safe option because of fear of pain and suboptimal quality of care during labor and vaginal birth [34]. To address women’s concerns, prenatal education on the benefits of vaginal delivery, pain-relief strategies, and midwifery training curricula were introduced and promoted in hospitals. A before-and-after retrospective study in East China suggested that implementation of epidural analgesia for labor increased the vaginal-delivery rate [35]. Meanwhile, a survey conducted in Southwest China demonstrated that women who attended an educational session by an anesthesiologist on epidural analgesia for labor preferred it to cesarean delivery, and that obstetricians can play an important role in such education [36]. Thus, childbirth training workshops for mothers or couples, continuous one-to-one intrapartum support, and pain management during labor are considered effective interventions for promoting vaginal delivery, and were included in the WHO recommendations on intrapartum care to achieve a positive childbirth experience [37, 38].

### Strengths and limitations

Our study had several strengths and limitations. First, the China Labor and Delivery Survey was a large, multicenter study involving secondary and tertiary hospitals covering most geographic regions in China. As approximately 90% of women gave birth in secondary or tertiary hospitals [39], and labor induction was almost always performed in the hospital, our data adequately represented Chinese labor inductions. Second, to our knowledge, this is the first large-scale epidemiological study to provide details on labor induction in China. We collected data on births from 24 weeks of gestation with a data-collection form that was previously used in studies by the WHO [40]. Third, the large sample size allowed us to compare the impacts of labor induction and prelabor cesarean delivery in low-risk women on maternal and neonatal outcomes, which might be difficult to conduct a clinical trial in real practice. Further investigations are warranted to evaluate the side-effects, costs and acceptability of these two procedures. Nonetheless, our study population was not a randomly selected sample of all hospitals that provide obstetric care in China and only included hospitals with more than 1000 deliveries per year. Therefore, our estimates may not represent all childbirths. In addition, as this was an observational study, we could not compare the effect of labor induction to that of expectant management on maternal and perinatal outcomes. Finally, maternal medical conditions, cervical status, and health resources may also influence the choice of induction method, which may impact the vaginal-delivery rate in women undergoing labor induction.

## Conclusion

In our survey, 18.4% of nulliparas and 10.2% of multiparas underwent labor induction in China from 2015 to 2016. A quarter of them had favorable cervixes before induction. A total of 72.9% of nulliparous and 86.6% of multiparous women who underwent labor induction achieved vaginal delivery. The proportion of prelabor cesarean delivery may contribute to regional differences in the labor-induction rate. Labor induction in low-risk women, compared with prelabor cesarean delivery, was not associated with severe maternal and neonatal morbidity.

## Supplementary Information

**Additional file 1:** **Table S1.** Associations between sociodemographic characteristics and cesarean delivery in women undergoing labor induction in China. **Table S2.** Gestational weeks at delivery and vaginal delivery rate among women undergoing labor induction in China. **Table S3.** Attempted mode of delivery by geographical regions in China. **Table S4.** Association of prelabor cesarean delivery rate and labor induction rate in China. **Table S5.** Comparison of maternal characteristics between low-risk women undergoing labor induction and prelabor cesarean delivery in China.

## Data Availability

Data is available upon reasonable request to the corresponding author.

## Abbreviations

WHO: World Health Organization
ACOG: American College of Obstetricians and Gynecologists
OR: Odds ratio
CI: Confidence interval
BMI: Body mass index
PROM: Premature rupture of membranes
SGA: Small for gestational age
