# Supplementary material for: Labor induction in China: a nationwide survey
Source: BMC Pregnancy Childbirth. 2022 Jun 1;22:463. doi: 10.1186/s12884-022-04760-6 (PMC9158355; doi:10.1186/s12884-022-04760-6)
Supplement: Supplementary file 1 — Additional file 1: Table S1. Associations between sociodemographic characteristics and cesarean delivery in women undergoing labor induction in China. Table S2. Gestational weeks at delivery and vaginal delivery rate among women undergoing labor induction in China. Table S3. Attempted mode of delivery by geographical regions in China. Table S4. Association of prelabor cesarean delivery rate and labor induction rate in China. Table S5. Comparison of maternal characteristics between low-risk women undergoing labor induction and prelabor cesarean delivery in China. [file 12884_2022_4760_MOESM1_ESM.docx]

**Table S1. Associations between sociodemographic characteristics and cesarean delivery in women undergoing labor induction in China.**

| Characteristics | Nulliparas | | |  | Multiparas | | |
| --- | --- | --- | --- | --- | --- | --- | --- |
|  | Weighted number of CS, N (%) * | Crude OR (95%CI) * | Adjusted OR (95%CI) *† |  | Weighted number of CS, N (%) * | Crude OR (95%CI) * | Adjusted OR (95%CI) *† |
| Maternal age (years) |  |  |  |  |  |  |  |
| < 25 | 38 216 (25.5) | 1.25 (0.75–2.09) | 1.35 (0.69–2.66) |  | 17 430 (19.1) | 1.19 (0.39–3.59) | 0.93 (0.27–3.27) |
| 25–29 | 99 591 (21.4) | Ref. | Ref. |  | 27 089 (16.6) | Ref. | Ref. |
| 30–34 | 61 800 (41.2) | 2.57 (1.38–4.79) | 2.94 (1.54–5.61) |  | 12 567 (8.3) | 0.45 (0.18–1.12) | 0.43 (0.15–1.21) |
| ≥ 35 | 14 027 (55.7) | 4.60 (1.85–11.5) | 5.38 (2.12–13.7) |  | 6327 (9.3) | 0.52 (0.21–1.25) | 0.57 (0.24–1.36) |
| Race |  |  |  |  |  |  |  |
| Han | 210 119 (27.3) | Ref. | Ref. |  | 61 830 (13.5) | Ref. | Ref. |
| Other | 3624 (18.0) | 0.58 (0.37–0.93) | 0.66 (0.42–1.05) |  | 1583 (10.3) | 0.73 (0.12–4.55) | 0.47 (0.03–6.48) |
| Education (years) |  |  |  |  |  |  |  |
| Less than high school (≤9) | 27 109 (23.0) | 0.80 (0.43–1.46) | 1.05 (0.53–2.10) |  | 34 896 (15.5) | 2.82 (0.90–8.87) | 1.57 (0.61–4.05) |
| High school (10–12) | 45 018 (30.0) | 1.14 (0.73–1.79) | 1.30 (0.94–1.81) |  | 18 122 (17.4) | 3.24 (1.05–9.99) | 2.08 (0.91–4.75) |
| College and above (>12) | 125 833 (27.4) | Ref. | Ref. |  | 7066 (6.1) | Ref. | Ref. |
| Social health insurance |  |  |  |  |  |  |  |
| Yes | 149 644 (27.4) | Ref. | Ref. |  | 37 962 (13.3) | Ref. | Ref. |
| No | 54 549 (27.1) | 0.99 (0.49–2.01) | 1.15 (0.48–2.76) |  | 24 640 (14.0) | 1.07 (0.36–3.12) | 1.13 (0.48–2.68) |
| BMI at delivery (kg/m^2^) |  |  |  |  |  |  |  |
| < 18.5 | 561 (51.5) | 4.38 (1.30–14.7) | 4.51 (1.09–18.6) |  | 57 (7.2) | 2.74 (0.27–27.6) | 3.79 (0.25–57.4) |
| 18.5–23.9 | 23 588 (19.5) | Ref. | Ref. |  | 1502 (2.8) | Ref. | Ref. |
| 24.0–27.9 | 82 225 (26.8) | 1.51 (1.23–1.86) | 1.42 (1.10–1.85) |  | 29 443 (16.4) | 6.98 (2.32–20.5) | 6.77 (2.29–20.0) |
| ≥ 28.0 | 85 131 (32.2) | 1.96 (1.30–2.94) | 1.77 (1.01–3.10) |  | 14 706 (11.7) | 4.68 (2.44–8.98) | 5.08 (1.81–14.2) |
| Unknown | 22 318 (23.0) | 1.23 (0.45–3.34) | 1.07 (0.33–3.45) |  | 17 705 (15.6) | 6.50 (2.86–14.8) | 3.76 (1.70–8.28) |
| Number of fetuses |  |  |  |  |  |  |  |
| Singleton | 213 365 (27.1) | Ref. | Ref. |  | 62 546 (13.3) | Ref. | Ref. |
| Multiple | 458 (15.1) | 0.48 (0.13–1.79) | 0.68 (0.23–1.97) |  | 867 (26.6) | 2.36 (0.24–23.0) | 8.47 (0.58–123.8) |
| Fetal presentation |  |  |  |  |  |  |  |
| Cephalic | 212 594 (27.1) | Ref. | Ref. |  | 63 041 (13.6) | Ref. | Ref. |
| Breech or other non-cephalic | 499 (16.9) | 0.55 (0.19–1.59) | 0.71 (0.24–2.10) |  | 276 (3.9) | 0.26 (0.05–1.27) | 0.20 (0.03–1.58) |
| Labor analgesia |  |  |  |  |  |  |  |
| Yes | 75 564 (30.2) | 1.26 (1.02–1.56) | 1.26 (0.98–1.62) |  | 30 985 (21.6) | 2.53 (1.15–5.55) | 2.84 (1.22–6.62) |
| No | 138 259 (25.6) | Ref. | Ref. |  | 32 428 (9.8) | Ref. | Ref. |
| Hospital level |  |  |  |  |  |  |  |
| Level 2 | 90 469 (28.9) | Ref. | Ref. |  | 47 910 (17.4) | Ref. | Ref. |
| Level 3 | 123 354 (25.8) | 0.86 (0.57–1.29) | 0.68 (0.43–1.08) |  | 15 503 (7.8) | 0.40 (0.14–1.17) | 0.44 (0.13–1.46) |
| Region |  |  |  |  |  |  |  |
| Central | 41 199 (25.9) | 0.82 (0.64–1.06) | 0.97 (0.68–1.39) |  | 30 165 (18.8) | 1.81 (0.50–6.60) | 1.82 (0.63–5.21) |
| East | 110 561 (29.8) | Ref. | Ref. |  | 20 969 (11.3) | Ref. | Ref. |
| North | 14 417 (28.0) | 0.91 (0.64–1.30) | 0.97 (0.62–1.51) |  | 1882 (14.0) | 1.28 (0.37–4.37) | 1.95 (0.61–6.26) |
| Northeast | 3029 (18.3) | 0.53 (0.30–0.92) | 0.60 (0.25–1.42) |  | 873 (12.8) | 1.16 (0.20–6.71) | 1.76 (0.31–9.83) |
| Northwest | 5823 (8.2) | 0.21 (0.08–0.54) | 0.20 (0.11–0.38) |  | 2127 (6.9) | 0.59 (0.06–5.70) | 0.53 (0.03–8.22) |
| South | 22 943 (28.2) | 0.92 (0.65–1.31) | 0.91 (0.50–1.65) |  | 3773 (6.6) | 0.56 (0.12–2.63) | 0.42 (0.07–2.46) |
| Southwest | 15 850 (39.9) | 1.57 (1.10–2.24) | 2.14 (1.39–3.29) |  | 3623 (18.1) | 1.73 (0.51–5.85) | 2.65 (0.98–7.14) |

BMI, body mass index; OR, odds ratio; CI, confidence interval.

*PROC SURVEYFREQ procedure was used to calculate the frequencies, adjusting for sampling weight and clustering of births within hospitals.

†PROC SURVEYLOGISTIC procedure was used to evaluate the association between maternal characteristics and cesarean section in women undergoing labor induction, compared with women induced without such characteristics. We adjusted for maternal age, race, insurance status, education level, BMI at delivery, number of fetuses, fetal presentation, labor analgesia, hospital location and hospital level.

**Table S2. Gestational weeks at delivery and vaginal delivery rate among women undergoing labor induction in China.**

|  | Gestational weeks at delivery (weeks), median (IQR) * | Vaginal delivery rate, % (95%CI) * | |
| --- | --- | --- | --- |
|  |  | Nulliparas | Multiparas |
| Maternal complications |  |  |  |
| Chronic hypertension | 39.7 (38.1–39.7) | 71.1 (52.5–89.8) | 89.7 (70.7–100.0) |
| Gestational hypertension | 39.6 (38.6–40.3) | 66.1 (48.4–83.8) | 94.3 (89.2–99.5) |
| Preeclampsia/eclampsia | 37.7 (35.6–39.0) | 78.4 (60.6–96.2) | 89.1 (75.5–100.0) |
| Diabetes mellitus | 39.6 (38.1–40.4) | 60.5 (40.8–80.1) | 87.3 (68.2–100.0) |
| Gestational diabetes | 39.9 (38.9–40.3) | 68.7 (62.8–74.7) | 96.1 (91.0–100.0) |
| Advanced maternal age | 39.7 (37.4–40.3) | 44.3 (24.3–64.4) | 90.7 (85.0–96.3) |
| Obesity | 40.0 (39.0–40.6) | 67.8 (63.4–72.2) | 88.3 (83.2–93.3) |
| Other conditions | 40.3 (39.4–40.4) | 81.0 (67.4–94.6) | 94.7 (87.6–100.0) |
| Fetal indications |  |  |  |
| SGA (< 10^th^ percentile) | 40.0 (37.7–40.6) | 83.3 (75.4–91.3) | 94.8 (89.7–99.9) |
| Suspected macrosomia | 40.3 (39.4–41.0) | 61.8 (56.1–67.6) | 91.1 (86.4–95.9) |
| Abnormal antenatal testing results | 40.1 (39.3–40.7) | 58.7 (45.8–71.6) | 31.4 (7.6–55.1) |
| Antepartum stillbirth | 29.0 (26.1–34.6) | 99.1 (97.9–100.0) | 99.4 (98.5–100.0) |
| Fetal anomaly | 32.6 (27.9–39.9) | 90.9 (79.7–100.0) | 99.3 (98.2–100.0) |
| PROM | 39.0 (37.4–39.9) | 72.7 (67.7–77.8) | 89.0 (82.4–95.7) |
| Late-term and post-term pregnancies | 41.3 (41.0–41.6) | 70.0 (65.9–74.2) | 74.0 (58.2–89.8) |
| Uterine scar | 36.9 (32.0–39.6) | 0 | 87.4 (74.8–100.0) |
| Nonmedically indicated | 39.9 (39.0–40.3) | 76.5 (69.1–83.9) | 87.4 (73.8–100.0) |

SGA, small for gestational age; PROM, premature rupture of membrane; IQR, interquartile range; CI, confidence interval.

*PROC SURVEYFREQ procedure was used to calculate the frequencies, adjusting for sampling weight and clustering of births within hospitals.

**Table S3. Attempted mode of delivery by geographical regions in China.**

|  | Attempted vaginal delivery | | Planned cesarean delivery | | Unknown | Total |
| --- | --- | --- | --- | --- | --- | --- |
|  | Spontaneous labor, N (%) * | Labor induction, N (%) * | Cesarean delivery without indications, N (%) * | Cesarean delivery with indications, N (%) * | Number of women, N (%) * | Number of women, N (%) * |
| Nulliparous |  |  |  |  |  |  |
| Central | 349 846 (48.6) | 158 934 (22.1) | 36 481 (5.1) | 168 144 (23.4) | 6240 (0.9) | 719 645 (16.8) |
| East | 885 627 (48.8) | 370 904 (20.4) | 92 294 (5.1) | 464 073 (25.6) | 2307 (0.1) | 1 815 204 (42.3) |
| North | 142 523 (57.7) | 51 557 (20.9) | 4500 (1.8) | 47 800 (19.4) | 636 (0.3) | 247 015 (5.8) |
| Northeast | 153 651 (57.4) | 16 570 (6.2) | 19 481 (7.3) | 77 929 (29.1) | 0 (0) | 267 630 (6.2) |
| Northwest | 155 132 (61.3) | 71 201 (28.1) | 883 (0.3) | 25 515 (10.1) | 335 (0.1) | 253 066 (5.9) |
| South | 393 023 (65.0) | 81 393 (13.5) | 17 273 (2.9) | 111 326 (18.4) | 1421 (0.2) | 604 437 (14.1) |
| Southwest | 221 662 (57.7) | 39 694 (10.3) | 34 754 (9.1) | 87 665 (22.8) | 163 (0.1) | 383 938 (8.9) |
| Total | 2 301 464 (53.6) | 790 253 (18.4) | 205 665 (4.8) | 982 454 (22.9) | 11 101 (0.3) | 4 290 936 (100.0) |
| Multiparous |  |  |  |  |  |  |
| Central | 588 125 (45.5) | 160 826 (12.4) | 18 868 (1.5) | 522 014 (40.4) | 3057 (0.2) | 1 292 890 (27.9) |
| East | 759 600 (45.2) | 185 320 (11.0) | 26 092 (1.6) | 709 829 (42.2) | 1398 (0.1) | 1 682 239 (36.3) |
| North | 67 767 (48.5) | 13 426 (9.6) | 1561 (1.1) | 56324 (40.3) | 557 (0.4) | 139 635 (3.0) |
| Northeast | 85 574 (53.5) | 6801 (4.2) | 4253 (2.7) | 63 467 (39.6) | 0 (0) | 160 093 (3.5) |
| Northwest | 74 925 (59.5) | 30 649 (24.4) | 243 (0.2) | 19 730 (15.7) | 277 (0.2) | 125 824 (2.7) |
| South | 535 159 (63.9) | 56 844 (6.8) | 8891 (1.1) | 235 387 (28.1) | 1679 (0.2) | 837 961 (18.1) |
| Southwest | 182 569 (46.0) | 20 000 (5.0) | 12 184 (3.1) | 181 389 (45.8) | 323 (0.1) | 396 466 (8.6) |
| Total | 2 293 719 (49.5) | 473 866 (10.2) | 72 092 (1.6) | 1 788 141 (38.6) | 7290 (0.2) | 4 635 107 (100.0) |

*PROC SURVEYFREQ procedure was used to calculate the frequencies, adjusting for sampling weight and clustering of births within hospitals.

**Table S4. Association of prelabor cesarean delivery rate and labor induction rate in China.**

|  | Nulliparas | |  | Multiparas | |
| --- | --- | --- | --- | --- | --- |
|  | Labor induction (%) * | |  | Labor induction (%) * | |
|  | β (95% CI) | *P* value |  | β (95% CI) | *P* value |
| Planned cesarean delivery (%) | –0.34 (–0.51 to –0.16) | <0.001 |  | –0.12 (–0.21 to –0.03) | 0.008 |
| Cesarean delivery without indications (%) | –0.57 (–0.92 to –0.22) | 0.002 |  | –0.16 (–0.66 to 0.34) | 0.522 |

CI, confidence interval.

*Linear regression analysis was used to assess the association between prelabor cesarean delivery rate and labor induction rate in each hospital, adjusting for sampling weight, hospital level and hospital location.

**Table S5. Comparison of maternal characteristics between low-risk women undergoing labor induction and prelabor cesarean delivery in China.**

|  | Labor induction  (n=382 547) | Prelabor cesarean delivery (n=506 076) |
| --- | --- | --- |
| Maternal age (years), N (%) * |  |  |
| < 25 | 69 441 (18.2) | 127 264 (25.2) |
| 25–29 | 201 808 (52.8) | 237 325 (47.0) |
| 30–34 | 92 581 (24.2) | 94 604 (18.7) |
| ≥ 35 | 18 176 (4.8) | 45 803 (9.1) |
| Race, N (%) * |  |  |
| Han | 373 725 (97.7) | 495 714 (98.0) |
| Other | 8822 (2.3) | 10 363 (2.0) |
| Education (years), N (%) * |  |  |
| Less than high school (≤9) | 92 883 (26.5) | 149 067 (34.0) |
| High school (10–12) | 74 182 (21.1) | 98 675 (22.5) |
| College and above (>12) | 183 624 (52.4) | 191 148 (43.6) |
| Social health insurance, N (%) * |  |  |
| Yes | 243 301 (68.8) | 306 232 (67.4) |
| No | 110 088 (31.2) | 147 827 (32.6) |
| BMI at delivery (kg/m^2^), N (%) * |  |  |
| < 18.5 | 752 (0.2) | 492 (0.1) |
| 18.5–23.9 | 51 153 (13.4) | 59 433 (11.7) |
| 24.0–27.9 | 151 808 (39.7) | 202 052 (39.9) |
| ≥ 28.0 | 111 598 (29.2) | 174 383 (34.5) |
| Unknown | 67 236 (17.5) | 69 716 (13.8) |
| Parity, N (%) * |  |  |
| 0 | 257 145 (67.2) | 366 975 (72.5) |
| ≥ 1 | 125 402 (32.8) | 139 101 (27.5) |
| Number of fetuses, N (%) * |  |  |
| Singleton | 380 186 (99.4) | 497 979 (98.4) |
| Multiple | 2360 (0.6) | 8097 (1.6) |
| Hospital level, N (%) * |  |  |
| Level 2 | 191 745 (50.1) | 271 950 (53.7) |
| Level 3 | 190 802 (49.9) | 234 126 (46.3) |
| Gestational weeks at delivery (weeks), median (IQR) | 39.9 (39.0–40.3) | 39.1 (38.4–40.0) |
| Birthweight (g), median (IQR) | 3350 (3150–3600) | 3300 (3050–3600) |

BMI, body mass index; IQR, interquartile range.

*PROC SURVEYFREQ procedure was used to calculate the frequencies, adjusting for sampling weight and clustering of births within hospitals.

Low-risk women were women with term pregnancies and without the following conditions: chronic hypertension, diabetes mellitus, thyroid disease, renal disease, autoimmune disease, heart disease, gestational hypertension, preeclampsia/eclampsia, gestational diabetes, cholestasis, SGA, suspected macrosomia, abnormal antenatal testing results, antenatal stillbirth, fetal anomaly, breech or other non-cephalic presentation, PROM, late-term or post-term pregnancy, uterine scar, placental abruption, placenta previa, and prolapse of the cord.
